# Whole-Genome Sequencing Data Reveal New Loci Affecting Milk Production in German Black Pied Cattle (DSN)

**DOI:** 10.3390/genes14030581

**Published:** 2023-02-25

**Authors:** Paula Korkuć, Guilherme B. Neumann, Deike Hesse, Danny Arends, Monika Reißmann, Siham Rahmatalla, Katharina May, Manuel J. Wolf, Sven König, Gudrun A. Brockmann

**Affiliations:** 1 Albrecht Daniel Thaer-Institute for Agricultural and Horticultural Sciences, Animal Breeding and Molecular Genetics, Humboldt-Universität zu Berlin, Invalidenstr. 42, 10115 Berlin, Germany; 2 Department of Applied Sciences, Northumbria University, Ellison PI, Newcastle upon Tyne NE1 8ST, UK; 3 Institute of Animal Breeding and Genetics, Justus-Liebig-Universität Gießen, Ludwigstr. 21, 35390 Gießen, Germany

**Keywords:** GWAS, *MGST1*, alpha S1-casein, serotonin receptor, lactation, milk yield, fat content, protein content, *GNG2*, *TNKS*, *TLE4*

## Abstract

German Black Pied (DSN) is considered an ancestral population of the Holstein breed. The goal of the current study was to fine-map genomic loci for milk production traits and to provide sequence variants for selection. We studied genome-wide associations for milk-production traits in 2160 DSN cows. Using 11.7 million variants from whole-genome sequencing of 304 representative DSN cattle, we identified 1980 associated variants (−log_10_(*p*) ≥ 7.1) in 13 genomic loci on 9 chromosomes. The highest significance was found for the *MGST1* region affecting milk fat content (−log_10_(*p*) = 11.93, MAF = 0.23, substitution effect of the minor allele (ß_MA_) = −0.151%). Different from Holstein, *DGAT1* was fixed (0.97) for the alanine protein variant for high milk and protein yield. A key gene affecting protein content was *CSN1S1* (−log_10_(*p*) = 8.47, MAF = 049, ß_MA_ = −0.055%) and the *GNG2* region (−log_10_(*p*) = 10.48, MAF = 0.34, ß_MA_ = 0.054%). Additionally, we suggest the importance of *FGF12* for protein and fat yield, *HTR3C* for milk yield, *TLE4* for milk and protein yield, and *TNKS* for milk and fat yield. Selection for favored alleles can improve milk yield and composition. With respect to maintaining the dual-purpose type of DSN, unfavored linkage to genes affecting muscularity has to be investigated carefully, before the milk-associated variants can be applied for selection in the small population.

## 1. Introduction

The dual-purpose cattle breed German Black Pied (DSN, German: “Deutsches Schwarzbuntes Niederungsrind”) is an endangered breed from Germany, with currently around 2500 herdbook cows [1]. Since DSN cows produce about 2500 kg less milk compared to Holstein cows, they were almost entirely replaced by Holstein in the 1960s and 1970s [2]. The DSN breed is maintained as a genetic reserve due to its beneficial properties, such as high milk fat and protein content (4.3% and 3.7%, respectively), their relation to Holstein as an ancestral breed [3], and their high genetic diversity despite the small population size [4]. Currently, DSN breeders are subsidized by the German government to compensate for the low milk yield. For maintaining DSN long-term, it is crucial to improve its milk production while keeping its dual-purpose character and breed-specific characteristics.

Genetic information for this small endangered population is needed to support its conservation and further development. Genome-wide association studies (GWASs) have been performed for milk production [5], clinical mastitis [6], endoparasite resistance and fertility [7]. These GWASs used genotypes from the Illumina BovineSNP50 BeadChip that contains around 34 K informative SNPs for DSN.

All commercially available bovine SNP chips contain SNPs that are biased towards cosmopolitan breeds. As those breeds and not the local breeds contributed to the chip design, the commercial chips do not accurately depict specific mutations and linkage disequilibria of local breeds such as DSN [8]. As a consequence, only a comparably small portion of SNPs (approximately 63%) on commercial SNP chips is informative for DSN [9]. More importantly, potentially functional causal mutations segregating the DSN breed are almost entirely absent. Furthermore, most SNP chips hardly target SNP-rich regions, since the probe design interferes with neighboring SNPs [10]. Therefore, a DSN-specific SNP chip was developed, which contains 182 K sequence variants that are highly informative, in particular for functional SNPs and SNPs unique to DSN [9].

Using high-density sequence information allows a fine-grained view of the whole genome, detection of associations with DSN-specific variants, and, in particular, the identification of variation in associated genes. Only the most recent studies in DSN included genotypes obtained from the customized DSN200K SNP chip and imputed whole-genome sequencing (WGS) data [11,12]. 

In the previous GWAS for milk production traits with 1490 DSN cows and 34 K informative SNPs of the Illumina BovineSNP50 BeadChip, we identified 41 significant SNPs on 17 chromosomes [5]. In the current study, we expanded our previous GWAS using a larger population and a saturated marker density of 11.7 million variants, as genotypes from the DSN200K SNP chip and the Illumina BovineSNP50 BeadChip were imputed to WGS level using the WGS data of 304 DSN animals [13]. We expected a higher significance and refined resolution of previously associated loci and, in particular, the identification of additional loci. The additional loci would result from the information on whole-genome variants which were not considered on the commercial SNP chip used in the previous study.

## 2. Materials and Methods

### 2.1. Population

We obtained genotypic and phenotypic data of 2598 DSN cows representing almost the whole DSN population. For the GWAS, we required that at least 20 cows per farm, per sire and per birth year were available in order to control environmental influences. Thus, analyses were performed with 2160 cows which were kept on eight farms in Germany. The cows were born between 2007 and 2017 and descended from 37 sires. Pedigree data were obtained from the breeding company RBB Rinderproduktion Berlin-Brandenburg GmbH.

### 2.2. Genotypes

**Figure 1:**
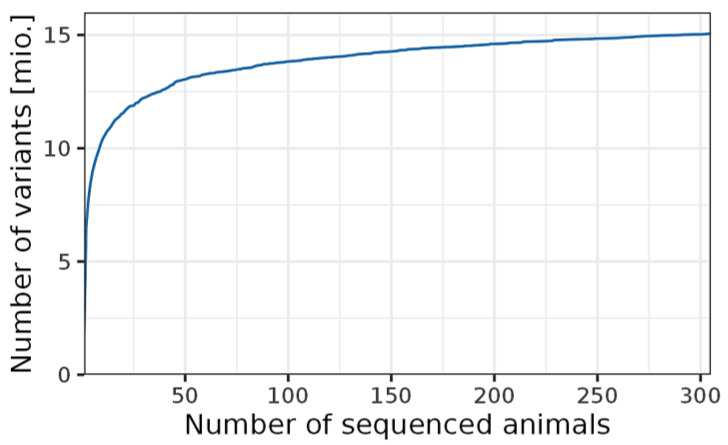
Number of identified variants per number of sequenced animals.

Genotypes of the 2160 DSN cows with available phenotypic data for GWAS were available from Illumina BovineSNP50 chips (1501 DSN cows) [5], custom DSN200K SNP chips (478 DSN cows) [9], and whole-genome sequencing (181 DSN cows) [9]. The DSN200K chip was designed with Axiom^®^ myDesign TG Array technology of Thermo Fisher Scientific Inc. (Waltham, MA, USA) and targeted 182 K sequence variants. For imputation, WGS data were available for 304 DSN cattle (181 cows which were used for GWAS, 76 additional cows and 47 bulls) [9]. Genotypes from Illumina BovineSNP50 or DSN200K SNP chips were imputed directly to the positions of the WGS data with Beagle v5.1 [13,14]. With regard to the size of the reference panel for imputation, we tested how much additional variation in the DSN population was found by subsequently adding the WGS data of additional animals (Figure 1). This followed a saturation curve. After reaching a certain number of animals, adding more animals to the reference panel did not significantly increase the number of newly identified variations. Based on this, we believe that having 304 DSN individuals in the reference panel for imputation was sufficient to capture the majority of variants in the investigated DSN population. Imputed WGS data counted 15,055,053 variants. Filtering for biallelic variants was performed with a minor allele frequency (MAF) of ≥5% and an SNP call rate of ≥90% using vcftools v0.1.14 [15], and resulted in 11,770,842 variants which were available for GWAS. Genomic positions of variants refer to the *Bos taurus* genome version ARS-UCD1.2 [16]. 

### 2.3. Phenotypes

Data regarding 305-day milk performance were obtained from the breeding company in March 2022 containing data for the years 2009 to 2021. Traits included milk, fat and protein yield in kilograms (milk kg, fat kg, protein kg) for the first three lactations (LA1, LA2, LA3) considering only full lactations (≥270 days in milk). Fat and protein content (fat %, protein %) were calculated by dividing the fat or protein yield by milk yield of the respective lactation. The lactation mean (LAm) was calculated when data of the respective trait were available in all three lactations. Values outside the mean ± 3 standard deviations of each trait were removed. Up to 1991, 1610, 1262 and 1010 DSN cows had milk performance data available in LA1, LA2, LA3 and LAm, respectively.

### 2.4. Genome-Wide Association Studies (GWASs)

GWASs were performed with multiple linear regression models implemented in the R language for statistical computing using the base packages [17]. The model for testing the additive effect of each SNP for each trait *Y* included fixed effects for population stratification *ps,* farm *f*, sire *s*, birth year *by*, birth season *bs*, calving year *cy*, calving season *cs* and age at first calving in days *ac*, together with the SNP genotype *gt* and the residual error *e:*

(1) $$Y=ps+f^{*}+s^{*}+by^{*}+bs^{*}+cy^{*}+cs^{*}+ac^{*}+gt+e$$

Population stratification was estimated using the pairwise population concordance test (parameters --cluster and --ppc) implemented in PLINK v1.9 [18] and the identity-by-state matrix of all 2160 DSN cows. The *p*-value cut-off of 1 × 10^−5^ resulted in 58 clusters of relatedness. Fixed effects marked with an asterisk (“*”) were included in the model only when the difference in Akaike information criterion (ΔAIC) between the null model (*Y = ps*) and the null model extended with one of the other fixed effects (*Y = ps + fixed effect*) was ≤−10 (Supplementary Table S1). The heritability of traits was calculated using GCTA software v1.93.2 [19] by performing an REML (restricted maximum likelihood) analysis on the GRM (genomic relationship matrix).

### 2.5. Significance Threshold and QTL Definition

Calculating the inflation factor of *p*-values from GWAS for all traits, we observed high average inflation factors λ on autosomes (1.51 ± 0.20 SD) as well as on chromosome X (2.09 ± 0.51 SD) (Supplementary Figure S1). The effective population size of DSN was low with N_eff_ = 107 [1]. Hence, DSN had a highly structured population, so we expected a certain inflation of *p*-values. To reduce the number of false positive associations with each trait, we corrected *p*-values to λ = 1.2 for autosomes and chromosome X, separately. 

Significance thresholds were set with Bonferroni correction, dividing the α-level by the number of independent variants. The number of independent tests was estimated using linkage disequilibrium (LD)-based pruning with PLINK v1.9 [17]. The parameter indep-pairwise was used with a window size of 500, a step size of 100, and an r^2^ value of 0.6, resulting in 642,791 independent variants. Thresholds for highly significant (*p* < 0.01), significant (*p* < 0.05) and suggestive (*p* < 0.1) associations were divided by the number of independent variants corresponding to −log_10_(*p*) ≥ 7.8, ≥7.1 and ≥6.8, respectively. Quantitative trait locus (QTL) regions were coarsely defined by grouping associated variants (*p* < 0.1) within ± 2.5 Mb on a chromosome across all traits. Regions with fewer than three associated variants were discarded from further analysis. Afterwards, QTL regions were defined around each of the top SNPs by taking all variants above the suggestive threshold for significance (*p* < 0.1) that were not more than 500 kb apart. For visualization, we added 100 kb up- and downstream to the QTL region.

### 2.6. QTL Annotation

Detected QTL regions as defined above were investigated for candidate genes. The *Bos taurus* gene annotation was downloaded from Ensembl release 106 [20]. A total of 21,880 protein-coding genes were considered. Genes in every QTL region were analyzed separately for gene ontology (GO) term enrichment using g:Profiler version e106_eg53_p16_65fcd97 in the R package gprofiler2 v0.2.1 [21]. The options for annotated genes and a g:SCS threshold of *p* < 0.1 for suggestive and *p* < 0.05 for significant GO term enrichments were used.

Variants in QTL regions were investigated for impact consequence on gene transcripts using variant effect predictor (VEP) from Ensembl [22]. For this, rare variants from the imputed WGS genotypes (MAF ≥ 1%) were also considered. Categories included variants with high, moderate or low impact. Additionally, the VEP predicted if missense variants were tolerant or deleterious using the SIFT algorithm [23]. Variants with an impact defined as intergenic, intron, non-coding transcript or non-coding transcript exon variant were not considered. Top SNPs located in intron or promoter regions were tested to examine whether they were located in transcription factor binding sites of vertebrates as obtained from JASPAR CORE database release 9 [24] using the R package TFBSTools v1.36.0 [25]. 

Identified QTL regions were investigated for an overlap with previously published associations and QTLs for the same trait from cattleQTLdb release 48 [26]. PubMed IDs of corresponding publications were obtained using R package easyPubMed v2.21. 

Figures were produced using the R package ggplot2 v3.4.0 [27]. Gene arrows and names were added to plots using the R packages gggenes v0.4.1 and ggrepel v0.9.2 [28,29]. *p*-values between genotype groups in SNP effect plots were estimated using pairwise *t*-tests and displayed using R package ggpubr v0.5.0 [30].

## 3. Results

### 3.1. Genomic Regions Associated with Milk Production Traits

We identified 13 significant and suggestive loci (−log_10_(*p*) > 6.8) on nine chromosomes (1, 3, 5, 6, 8, 10, 20, 21 and 27) for all investigated milk production traits except the single lactation traits milk yield in LA1, and fat and protein yields in LA2 (Table 1). In total, 1,980 sequence variants (733 highly significant, 707 significant and 540 suggestive) were associated with milk performance traits (Supplementary Table S2). Due to the high correlation between traits, some variants were associated with multiple traits. Correlations between yield traits were high within single lactations (r ≥ 0.77) and moderate across lactations (r ≥ 0.37) (Supplementary Table S3). Fat content as well as protein content were moderately correlated across lactations (r ≥ 0.41 and r ≥ 0.55, respectively). Within a lactation, fat and protein content had only a low correlation (r ≤ 0.27). The protein yield and content, as well as fat yield and content, were not correlated (r ≤ 0.08). Depending on the investigated lactation, the heritability for milk yield ranged between 0.37 and 0.42, for fat yields between 0.29 and 0.42, and for protein yields between 0.32 and 0.38 (Supplementary Table S4). The heritability for milk fat percentage in DSN ranged between 0.45 and 0.60 and for milk protein percentage between 0.59 and 0.72. These values are consistent with expected values for Holstein and other breeds [31,32,33].

Highly significantly associated genomic loci (−log_10_(*p*) ≥ 7.8) were identified for milk yield on chromosomes 1 and 8, for fat content on chromosome 5, for protein content on chromosomes 5, 6 and 10, for fat yield on chromosome 27 and for protein yield on chromosome 1. In the following, we explain only the most significant loci.

### 3.2. Most Significant Locus Affecting Milk Fat and Protein Content on Chromosome 5

**Figure 2:**
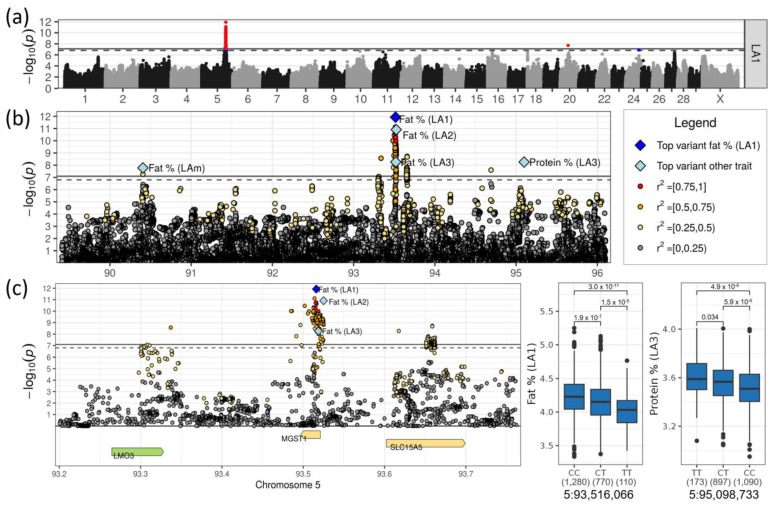
(**a**) Manhattan plot for milk fat content in LA1. Suggestive (*p* < 0.1, dashed line) or significant (*p* < 0.05, solid line) associations are highlighted in blue and red, respectively. (**b**) Regional association plot for the QTL on chromosome 5. (**c**) Zoomed regional association plot including genes and effect plots for top SNPs for milk fat content in LA1 and protein content in LA3. Genes containing variant(s) with low or moderate impacts on gene transcripts are colored green and yellow, respectively.

The most significant locus was identified for milk fat content on chromosome 5 (Table 1, Figure 2b). This effect was significant across all lactations whereas with the most significant effect found in LA1 in association with rs211210569 (−log_10_(*p*) = 11.93) at 93,516,066 bp. Its minor allele T, which segregated in DSN at a frequency of 0.23, reduced milk fat content in LA1 by 0.151% points. The allele effect was additive (Figure 2c). The top SNPs for lactations LA2, LA3 and LAm were located 9 kb downstream (5:93,525,076, rs207994397), 3 kb downstream (5:93,518,685, rs209372883) and 3.1 Mb upstream (5:90,406,099, rs134606936) of the top SNP in LA1, respectively. The negative effect of the minor allele of rs207994397 on fat content was highest in LA2 (β_MA_ = −0.226%).

The same locus also had a highly significant effect on protein content in LA3, with the top SNP rs41604619 at 95,098,733 bp (−log_10_(*p*) = 8.26, Table 1, Figure 2b). This SNP was located 1.5 Mb upstream of the top SNP for fat content in LA1. While the minor allele of the top SNP for fat content rs211210569 reduced the fat content, the minor allele T (MAF = 0.29) of the top SNP for protein content rs41604619 increased the protein content by 0.061% points. The top SNPs affecting milk fat and protein content were in low linkage disequilibrium (LD) of r^2^ = 0.08 (D’ = 0.80). 

The top SNP rs211210569 associated with fat content in LA1 was located in intron 1 out of three introns of the gene *MGST1* (microsomal glutathione S-transferase 1). In addition, sequence data of DSN revealed two missense variants in *MGST1* (5:93,497,602, novel; 5:93,509,514, rs210140457), both with tolerated impact on the protein function (Supplementary Table S5). 

Although *MGST1* harbored the most significant SNP, the neighboring genes *LMO3* (LIM domain only 3) and *SLC15A5* (solute carrier family 15 member 5) were also located in the highly associated region (Table 2). Local top SNPs were found in *LMO3* (5:93,337,092, rs109041635) as well as in *SLC15A5* (5:93,658,801, rs451608212). The top SNPs were linked to the key SNP in *MGST1* with r^2^ = 0.61 (D’ = 0.96) and r^2^ = 0.43 (D’ = 0.82), respectively. *LMO3* harbored one synonymous variant only and *SLC15A5* contained two tolerated (5:93,617,050, rs209784274; 5:93,650,729, rs136481676) and two deleterious missense variants (5:93,617,097, rs109333413; 5:93,627,362, rs211525134) (Supplementary Table S5). The deleterious missense variants caused amino acid changes at positions 247 and 291 of 571 amino acids in the protein. The amino acid changes were located between two transmembrane domains which belong to the “Proton-dependent oligopeptide transporter family” (Interpro: IPR000109, UniProtKB: F1N3P6_BOVIN). Moreover, *MGST1* was suggestively assigned to the biological process GO term “cellular response to lipid hydroperoxide” (GO:0071449) and *LMO3* was assigned to the “positive regulation of glucocorticoid receptor signaling pathway” (GO:2000324) (Supplementary Table S6). *SLC15A5* was involved in “transmembrane transport” (GO:0055085), but was not significant in the enrichment analysis.

### 3.3. Loci on Chromosome 1 and 8 Affect Milk Yield as Well as Fat and Protein Yields

**Figure 3:**
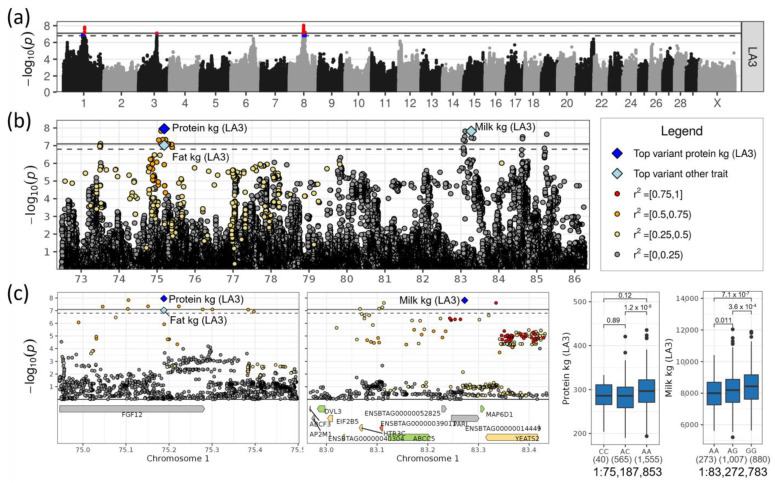
(**a**) Manhattan plot for milk yield in LA3. Suggestive (*p* < 0.1, dashed line) and significant (*p* < 0.05, solid line) associations are highlighted in blue and red, respectively. (**b**) Regional association plot for the QTL on chromosome 1. (**c**) Zoomed regional association plot including genes and effect plots for top SNPs for protein and milk yield in LA3. Genes containing variant(s) with low, moderate or high impact are colored green, yellow and orange, respectively, and are otherwise grey.

Loci on chromosomes 1 and 8 were highly significantly associated (−log_10_(*p*) >7.8) with milk yield and other yield traits (Table 1, Figure 3a,b). The locus on chromosomes 1 also had significant effects on the fat and protein yield, while the locus on chromosome 8 had a significant effect on the protein yield. 

The significant effects on chromosome 1 were all observed in LA3. The top SNP associated with milk yield was rs209578598 at 83,272,783 bp (−log_10_(*p*) = 7.82). Its minor allele A had a frequency of 0.36 and decreased the milk yield by 349 kg (Table 1, Figure 3c). In the same chromosomal region, rs379781684 at 75,187,853 bp was the top SNP affecting protein (−log_10_(*p*) = 7.96) and fat yield (−log_10_(*p*) = 7.03). The minor allele C had a frequency of 0.15 and decreased the protein yield by 16.8 kg and fat yield by 19.4 kg. The top two SNPs had a distance of 8.1 Mb. They were in low-to-moderate linkage in terms of the common LD estimators (r^2^ = 0.05, D’ = 0.41). 

*FGF12* (fibroblast growth factor 12) was the only gene residing in the top region for fat and protein yield of chromosome 1 and was, therefore, the most likely candidate gene (Table 2). The top SNP was in intron 3 of four introns of *FGF12*. No variants occurred in DSN that might have had an impact on the protein sequence of *FGF12* (Supplementary Table S5). 

The top region for milk yield of chromosome 1 (rs209578598, 1:83,272,783) contained seven genes (Table 2). The top SNP was in intron 4 of nine introns of the gene *PARL* (presenilin associated rhomboid like) (Supplementary Table S2). This SNP was in high LD with SNPs in *YEATS2* (YEATS domain containing 2), a gene that carries many additional SNPs, but its specific function is barely known. Interesting functional genes were *HTR3C* and ENSBTAG00000039011, which encode the 5-hydroxytryptamine (serotonin) receptor, family member C; however, ENSBTAG00000039011 was only predicted and could have simply been a gene duplication. Furthermore, *HTR3C* carried two missense variants (1:83,069,525, rs385043393, −log_10_(*p*) = 6.88; 1:83,064,588, rs381149322), and ENSBTAG00000039011 had two splice acceptor variants which had a high impact on gene transcripts (Supplementary Table S5). The receptor is necessary for the signaling of serotonin (REAC:R-BTA-112314), a neurotransmitter that is essential for the regulation of milk synthesis in the epithelium of the mammary gland (Supplementary Table S6). The gene *ABCC5* (ATP binding cassette subfamily C member 5) resided in the middle of the region associated with milk yield and contained three synonymous and one splice region variant (Supplementary Table S5). This gene is involved in tripeptide (or more precisely glutathione) transmembrane transporter activity (GO:0034634, GO:0042937) (Supplementary Table S6).

**Figure 4:**
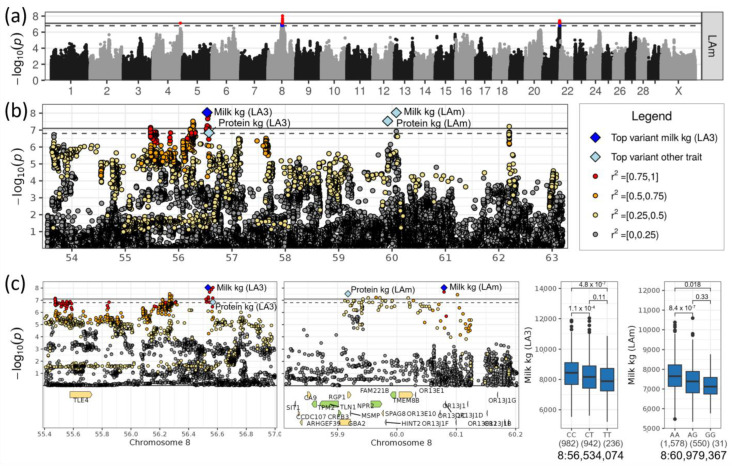
(**a**) Manhattan plot for milk yield in LAm. Suggestive (*p* < 0.1, dashed line) and significant (*p* < 0.05, solid line) associations are highlighted in blue and red, respectively. (**b**) Regional association plot for the QTL on chromosome 8. (**c**) Zoomed regional association plot including genes and effect plots for top SNPs for milk yield in LA3 and LAm. Genes containing variant(s) with low, moderate and high impacts are colored green, yellow and orange, respectively, and are otherwise grey.

On chromosome 8, the top SNP affecting milk yield in LA3 was rs385677618 at 56,534,074 bp (−log_10_(*p*) = 8.04) and in LAm rs797297575 at 60,079,367 bp (−log_10_(*p*) = 8.03, Table 1, Figure 4a,b). The minor alleles T of the top SNP for LA3 and G of the top SNP for LAm had frequencies of 0.33 and 0.14 and decreased the milk yield in LA3 by 346 kg and 280 kg, respectively. The same region showed an association with the protein yield in LA3 and LAm with the top SNP rs432948152 at 56,568,636 bp (−log_10_(*p*) = 6.84) and rs210911072 at 59,917,537 bp (−log_10_(*p*) = 7.53), respectively. The minor alleles of those two variants decreased the protein yield by 8.5 kg and 6.4 kg, respectively. The minor alleles were dominant (Figure 4c). These two SNPs, which were 3.5 Mb apart from each other, had a moderate linkage with each other (r^2^ = 0.23, D’ = 0.84).

The top SNPs for milk and protein yields (rs385677618 and rs432948152, respectively) were intergenic between *TLE4* (TLE family member 4, transcriptional corepressor) and the pseudogene ENSBTAG00000006294 (ATP synthase subunit f, mitochondrial pseudogene) (Table 2). Both genes were more than 500 kb away from the top SNP. *TLE4* was the only protein coding gene. The gene contained one tolerated missense variant in the DSN population (8:55,714,236, rs719817703, Supplementary Table S5). The gene *TLE4* was included in the pathway “Degradation of β-catenin by the destruction complex” (REAC:R-BTA-195253) belonging to the “Wnt signaling” pathways (Supplementary Table S6). 

The region around the second top SNP (rs797297575) on chromosome 8 at about 60 Mb comprised twelve different genes (Table 2), among which were four olfactory receptor genes which are involved in the “Olfactory transduction” pathway (KEGG:04740, Supplementary Table S5). The top SNP itself was located between the *OR13E10* (olfactory receptor family 13 subfamily E member 10) and *OR13J1C* (olfactory receptor family 13 subfamily J member 1C) genes, which are both less than 7 kb away. Among the olfactory receptor genes, *OR13E1* (olfactory receptor family 13 subfamily E member 1), *OR13E10* and *OR13J1F* (olfactory receptor family 13 subfamily J member 1F) had variants with high impacts on gene transcripts, including frameshift and stop-gained mutations, but also variants with moderate impact such as missense variants (Supplementary Table S5). Five additional genes had variants with moderate or even deleterious impacts: *GBA2* (glucosylceramidase β 2)*, RGP1* (RGP1 homolog, RAB6A GEF complex partner 1)*, SPAG8* (sperm associated antigen 8)*, TMEM8B* (transmembrane protein 8B) and *OR13J1C* (olfactory receptor family 13 subfamily J member 1C). 

### 3.4. Loci on Chromosomes 6 and 10 Affected Milk Protein Content

**Figure 5:**
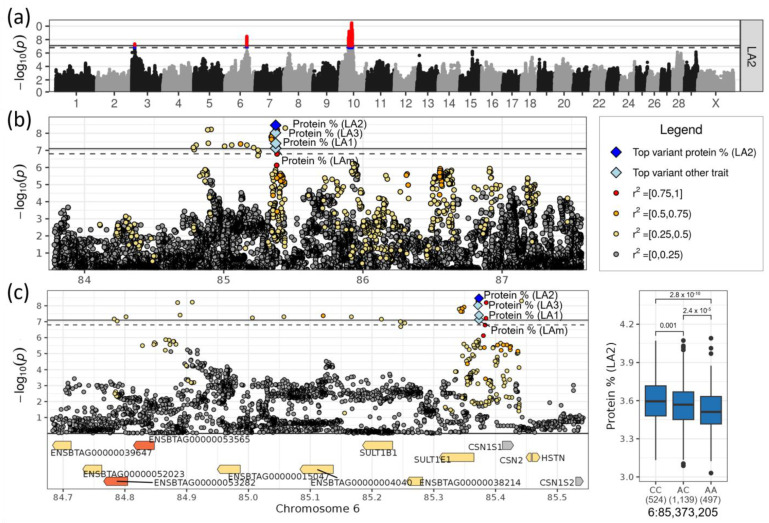
(**a**) Manhattan plot for milk protein content in LA2. Suggestive (*p* < 0.1, dashed line) and significant (*p* < 0.05, solid line) associations are highlighted in blue and red, respectively. (**b**) Regional association plot for the QTL on chromosome 6. (**c**) Zoomed regional association plot including genes and effect plot for top SNP for protein content in LA2. Genes containing variant(s) with low, moderate or high impact are colored green, yellow and orange, respectively, and are otherwise grey.

Loci on chromosomes 6 and 10 were highly significant associated, specifically with milk protein content (Table 1, Figure 5a). There were no effects observed for the other investigated traits. 

On chromosome 6, the top SNP rs378558630 at 85,373,205 bp was associated with protein content in LA1, LA2 and LAm with the highest significance in LA2(−log_10_(*p*) = 8.47, Table 1). The minor allele A had a frequency of 0.49 and decreased the protein content ranging from 0.035% points in LA1 to 0.055% points in LA2. The top SNP for LA3 rs382685419 was only 2 kb upstream of the other SNP (Figure 5b). Its minor allele T had a similar frequency (MAF = 0.50) and similar negative effect size, reducing the protein content by 0.054% points. The allele effects were additive (Figure 5c). The two SNPs were in high LD (r2 = 0.96, D’ = 1.0).

Although the whole significant region contained eight genes (Table 2), the top two SNPs were intergenic between *SULT1E1* (sulfotransferase family 1E member 1) and *CSN1S1* (α-S1 casein) (Figure 5c). *CSN1S1* was the only gene in the associated region that is encoded on the positive strand; all others were on the negative strand. The high density of highly significant SNPs around rs378558630 upstream of *CSN1S1* could affect the expression of *CSN1S1* and thereby regulate the milk protein content. The casein genes *CSN2* (β casein), *CSN1S2* (α-S2 casein) and *CSN3* (kappa casein) were close to but not directly within the associated region (Figure 5c). Near the top SNP, *SULT1E1* carried missense variants. *SULT1E1* and *SULT1B1* (Sulfotransferase Family 1B Member 1), which were up- and downstream of *CSN1S1*, were involved in sulfation processes and sulfotransferase (GO:0051923, GO:0008146) (Supplementary Table S6). Furthermore, the gene ENSBTAG00000053565 (UDP-glucuronosyltransferase 2C1) contained two variants with a high impact on its function including a stop-gained and a frameshift mutation (Supplementary Table S5); the protein contributed to the pathways “Steroid hormone biosynthesis” (KEGG:00140) and “Pentose and glucuronate interconversions” (KEGG:00040) (Supplementary Table S6).

**Figure 6:**
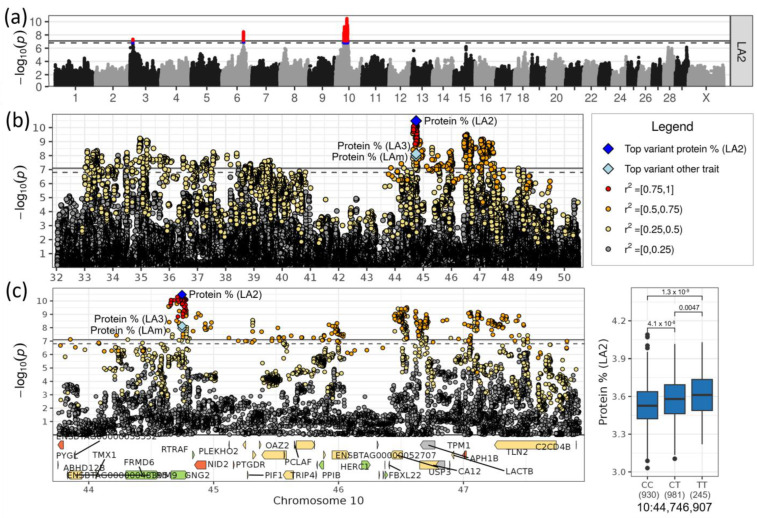
(**a**) Regional association plot for QTL for protein content on chromosome 10. (**b**) Zoomed regional association plot including genes and effect plot for top SNP for protein content in LA2. (**c**) Zoomed regional association plot including genes and effect plot for top SNP for protein content in LA2. Genes containing variant(s) with low, moderate or high impact are colored green, yellow and orange, respectively, and are otherwise grey.

On chromosome 10, the two SNPs rs211239920 at 44,746,907 bp (−log_10_(*p*) = 10.48) and rs208655317 at 44,746,980 bp (−log_10_(*p*) > 7.95), which were only 73 bp downstream of the first SNP, were highly significantly associated with protein content in LA2, LA3 and LAm, respectively (Table 1, Figure 6a). The minor allele T of the most significant SNP rs211239920 with a frequency of 0.34 increased the protein content by 0.054% in LA2, meaning the allele effect was additive (Figure 6b). The two top SNPs, rs211239920 and rs208655317, were intronic variants (intron 2 of three introns) of the gene *GNG2* (G protein subunit γ 2). The SNP rs211239920 was located in the putative transcription factor binding site of HMBOX1 (MA0895.1), a target for the Homeobox-containing protein 1. An additional 15 highly linked (r^2^ > 0.9) and highly associated SNPs (−log_10_(*p*) > 7.8) were in the *GNG2* gene region (Supplementary Table S2). The whole region underlying the top variant rs211239920 comprised 40 genes (Table 2). A total of 21 of those 40 genes comprised variants with a moderate impact on gene transcripts. The five genes ENSBTAG00000053552 (mTORC1-mediated signaling), *NID2* (nidogen 2), *PTGDR* (prostaglandin D2 receptor), ENSBTAG00000040590 and *TPM1* (tropomyosin 1) contained even variants with a high impact such as splice acceptors and frameshift variants.

### 3.5. A Locus on Chromosome 27 Affected Milk Fat Yield

**Figure 7:**
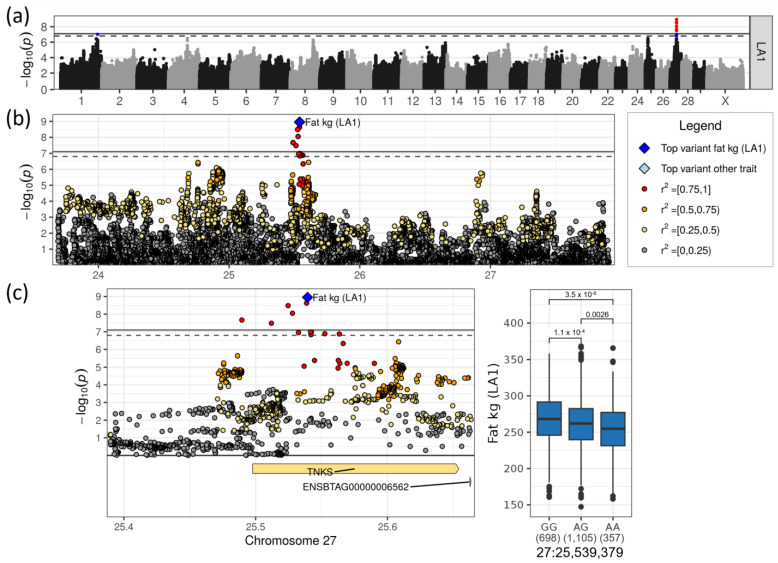
(**a**) Manhattan plot for fat yield in LA1. Suggestive (*p* < 0.1, dashed line) and significant (*p* < 0.05, solid line) associations were highlighted in blue and red, respectively. (**b**) Regional association plot for the QTL on chromosome 27. (**c**) Zoomed regional association plot including genes and effect plot for top SNP for fat yield in LA1. Genes containing variant(s) with moderate impact are colored yellow.

On chromosome 27, a highly significant QTL for milk fat yield in LA1 was found with rs42120938 as the top SNP at 25,539,379 bp (−log_10_(*p*) = 8.95, Table 1, Figure 7a,b). The minor allele A of this variant had an allele frequency of 0.42 and decreased the fat yield in LA1 by 9.4 kg. The allele effect was additive (Figure 7c). The top SNP was located in intron 2 of *TNKS* (tankyrase), a large gene consisting of 27 exons (Supplementary Table S2). Interestingly, *TNKS* carries a novel deleterious missense variant that segregates in DSN (27:25,625,081), but did not reach the significance threshold (Supplementary Table S5). The deleterious missense variant caused an amino acid change at position 825 of 1327 amino acids in the protein located between two ankyrin repeats, which are common protein–protein interaction motifs (Interpro:IPR002110, UniProtKB:E1B8R5_BOVIN). *TNKS* was assigned to the metabolic pathway “Degradation of AXIN” (REAC:R-BTA-4641257), which contributes to the “Wnt signaling” pathway (Supplementary Table S6). 

## 4. Discussion

In this study, we performed GWASs with milk performance data in a population of 2160 DSN cows using WGS data. Since the herdbook population of DSN is small, with only approximately 2500 cows in total, the population available for this association study was also small. Despite the limited power to find significant genomic loci in such a small population, it was possible to detect 1980 associated variants located in 13 loci on nine chromosomes. This was mainly possible due to the use of WGS data.

Some of the identified loci were pleiotropic, such as the locus on chromosome 5, where milk fat as well as protein content were affected. The loci on chromosomes 1 and 8 were associated with different yield traits, namely, milk, protein and fat yields on chromosome 1, and milk and protein yields on chromosome 8. The pleiotropy on chromosomes 1 and 8 contributed to the high correlations between the yield traits, especially within the same lactation.

The identification of different loci and effects for the same milk production trait across different lactations was partly due to the generally low and declining number of animals from lactation 1 to 3, and adventitious environmental effects, not all of which could be corrected for. As we examined “field data”, large individual differences due to environmental fluxes were expected. Besides these effects, genes necessary for the development of the mammary gland and for metabolic pathways contributing to milk production differed between the first and subsequent lactations. Genetic determinants driving differential development in different stages of life are well captured through GWASs, which examine every lactation separately. 

In our previous GWASs for milk production traits in DSN [5], where the sample size and number of SNP genotypes were smaller (1490 DSN cows, 36K SNPs from Illumina BovineSNP50), we identified 41 significantly associated SNPs located on 17 chromosomes. Not all of those loci could be confirmed in this study. Nevertheless, seven associated loci from this study and the previous one overlapped (on chromosomes 1, 3, 6, and 8); six loci were novel and have not shown an association before (on chromosomes 5, 10, 21 and 27). 

Consistent with the catalogue of published GWAS results obtained from the cattleQTLdb release 48, the *MGST1* locus identified in DSN cows for fat and protein content was reported for different Holstein populations and diverse other breeds such as Braunvieh, Fleckvieh, Normande and Montbéliarde [34,35,36,37]. Similarly, evidence was provided in numerous independent association studies for associations with the casein gene cluster on chromosome 6 for protein content [34,35,38,39], the locus on chromosome 10 for protein content [34,38,40,41], the locus on chromosome 20 for protein content [38,40,41,42] and the locus on chromosome 21 for milk yield [42]. A comprehensive list of 24 publications that reported loci for the same milk performance traits as presented here is available in Supplementary Table S7.

The most exciting result was the identification of the locus on chromosome 5 harboring *MGST1*, which affects milk fat content, while simultaneously missing an effect of *DGAT1* on chromosome 14 as the milk fat-producing gene with the highest effect in Holstein and other breeds. This finding was surprising for two reasons: Firstly, DSN is considered an ancestor population of Holstein, suggesting there should be an effect of *DGAT1* in DSN as well. However, *DGAT1* was found to be almost fixed for the Alanine protein variant of the K232A polymorphism in the investigated DSN population (frequency of 0.97), which is associated with higher milk and protein yield [43], and, therefore, cannot be associated with any trait. Secondly, because the impact of *MGST1* was high and had not been identified in our previous study with the commercial IlluminaBovineSNP50 BeadChip [5]. The identification of this locus was only possible using imputed DSN-specific whole-genome sequencing data [44].

As mentioned above, *MGST1* had been identified before as a candidate gene for milk fat content in numerous GWASs with different cattle breeds. Those studies could not fully rule out effects of the neighboring genes *LMO3* and *SLC15A5*. Although we also prioritize *MGST1* as the key candidate gene for milk fat content on chromosome 5, we also cannot rule out effects of the neighboring genes that showed smaller peaks of associated SNPs. Previously, a strong eQTL effect for *MGST1* expression in mammary tissue of dairy cattle supported the regulatory effect of *MGST1* on milk fat content [45]. This was also underlined by an enriched expression of *MGST1* in adipocytes in human breast (www.proteinatlas.org) [46]. It contributes to many metabolic pathways, among them the estrogen metabolism (www.proteinatlas.org). Knockout of *MGST1* in mice resulted in lower plasma cholesterol concentrations (www.mousephenotype.org) [47], which in turn is a marker for the negative energy balance in early lactating cows [48]. Therefore, *MGST1* might contribute to milk fat via the regulation of energy and/or fatty acids for the production of milk fat in the mammary gland. Nevertheless, the neighboring genes *LMO3* and *SLC15A5*, which showed smaller peaks of associated variants, remain interesting candidate genes. *SLC15A5* is responsible for protein transport through membranes and *LMO3* was shown to contribute to reprogramming of adipose tissue depots during obesity, thereby modulating nutrient homeostasis [49]. Transcripts of both genes were enriched in breast adipocytes (www.proteinatlas.org). 

On chromosome 1, two loci were associated with milk, protein and fat yield. In the locus affecting protein and fat yield, *FGF12* was the only candidate gene. *FGF12* has an impact on ribosome biogenesis [50] and, therefore, on the translation amount and efficiency, which in turn could directly influence the protein yield in milk and indirectly the milk fat yield. Among the other genes in the second locus 8.1 Mb downstream, *YEATS2, ABCC5* and *HTR3C*/ENSBTAG00000039011 are interesting candidate genes. *YEATS2* expression is enriched in breast myoepithelial cells (www.proteinatlas.org) and *ABCC5* was shown to influence body fat mass, probably by regulating GLP-1 (glucagon-like peptide 1) [51]. The serotonin receptor *HTR3C*/ENSBTAG00000039011 is necessary for the signaling of serotonin, a neurotransmitter that is essential for the regulation of milk synthesis in the epithelium of the mammary gland. However, in general, no gene can be prioritized based on the current mapping resolution. 

On chromosome 8, the whole region between 55 and 60 Mb was significantly associated with milk and protein yield. Although two different top SNPs with a distance of about 3.5 Mb were mapped for the yield traits in LA3 and LAm, we would not expect two genes to be underlying the statistical finding in this region. Even though *TLE4* is the only candidate gene close to the top SNP for milk and protein yield in LA3, the other genes in the whole region cannot be excluded. *TLE4* remains interesting as a transcriptional corepressor, as it has been linked to obesity in both mice and humans before [52]. Close to the second-ranked SNP were several SNPs carrying functional mutations which might be functionally interesting; however, prioritization of a key candidate gene was impossible on the basis of the current data. Among the interesting genes were *GBA2* (Glucosylceramidase β 2) and *HINT2* (Histidine triad nucleotide binding protein 2), which are involved in lipid metabolism (www.proteinatlas.org), and *NPR2* (Natriuretic peptide receptor 2), which is enriched in breast fibroblast (www.proteinatlas.org). 

A highly significant locus was identified on chromosome 6 in the region of the casein cluster, with *CSN1S1* as the closest and most likely candidate gene. This association was found in our previous GWAS [5]. The encoded α-S1 casein comprised 40% of the casein fraction in bovine milk [53]. This casein protein is important for the capacity of milk to transport calcium phosphate (www.proteinatlas.org). Polymorphisms in the regulatory region of *CSN1S1* contribute to differences in the transcription level and, therefore, to the amount of α-S1 casein produced, which is known to influence milk protein as well as milk fat content and milk properties [54,55,56]. The genetic architecture of the casein gene cluster of DSN in comparison to other breeds was investigated in detail [57]. 

The locus on chromosome 10 associated with protein content was, with 3.98 Mb and 40 genes, the biggest of all identified loci in this study. As a result of the high number of associated variants and genes in this region, no gene could be prioritized. Among the interesting genes with respect to mutations and functions were *GNG2* (G protein subunit γ 2)*, TRIP4* (Thyroid hormone receptor interactor 4) and *LACTB* (Lactamase β). The most significant SNPs were located in *GNG2*. As a G protein subunit, *GNG2* is involved in signaling mechanisms across membranes. *GNG2* is enriched in breast adipocytes (www.proteinatlas.org). *TRIP4* may act in energy partitioning, thereby ensuring sufficient energy for milk production, and *LACTB* is involved in lipid metabolism (www.proteinatlas.org). 

*TNKS* was the only candidate gene for the milk fat yield locus on chromosome 27. *TNKS* occurs in many tissues and it was found in various cell types of the brain. A knockout mouse study (www.mousephenotype.org) linked this gene to reduced body fat content and lower levels of blood glucose, blood protein and blood cholesterol, most likely by regulating vesicle trafficking and modulating the localization of GLUT4 [58]. 

As already mentioned above, the high quality of imputed DSN-specific WGS data was the key to the findings of this study. In order to define the population substructure, e.g., in an identity–state matrix, the WGS data provided the most comprehensive information of DSN-specific genomic variants between individuals. A downside of the usage of WGS data was the higher significance threshold caused by the higher number of variants tested. Therefore, the *p*-values thresholds were more stringent and a higher number of animals was needed to reach the power to identify associations.

## 5. Conclusions

This GWAS using imputed WGS data for 2160 DSN identified 13 loci on nine chromosomes associated with milk production traits in different lactations in dual-purpose DSN cattle. The resolution of WGS data helped to pinpoint associated genomic loci and the underlying genes, despite the limitation that the highest possible sample size of the investigated DSN population was still relatively small. Although DSN is considered an ancestor breed of Holstein, the major gene affecting milk fat content in DSN was *MGST1*, while *DGAT1* was the major gene affecting fat content and milk yield in Holstein cattle, which is fixed for the alanine protein variant for high milk yield in DSN. Additionally, we prioritized the following genes upon presence of highly significant SNPs located in or close to these genes, the putative function of SNPs and the function of affected genes: *FGF12* for protein and fat yield, *HTR3C* for milk yield, *CSN1S1* and *GNG2* for protein content, *TLE4* for milk and protein yield and *TNKS* for milk and fat yield. 

Since the DSN population was small, the development of a scheme which utilizes the obtained information for genomic selection is challenging. For increasing a learning population, data of several generations of animals has to be collected. Nevertheless, most significant SNPs contributing to variation in DSN cattle could be used to select for high milk yield and content traits to improve the economic merit of the animals on the market. However, this requires additional information on potentially linked gene variants that would undermine the breeding goals. To prevent negative effects on other important production traits or DSN characteristic traits, such as carcass and meat, conformation, fertility and health, additional studies with those traits have to be performed. The results of this study are a basis for further genetic analysis to identify causal genes and variants that affect milk traits in DSN directly.

## Figures and Tables

**Table 1:** Top SNPs associated with milk production traits in DSN cows. For each investigated trait, associated SNPs (rsID) are listed with chromosomal (Chr) positions (Position) with regard to the ARS-UCD1.2 genome assembly. MA = minor allele, MAF = minor allele frequency, β_MA_ = allele substitution effect of MA and its standard error (SE). Test statistics with −log_10_(*p*) ≥ 6.8, ≥7.1 and ≥7.8 were suggestive, significant and highly significant, respectively.

Trait (Lactation)	rsID	Chr	Position	MA	MAF	β_MA_	SE(β_MA_)	−log_10_(*p*)
Protein kg (LA3)	rs379781684	1	75,187,853	C	0.15	−16.8	2.7	7.96
Fat kg (LA3)	rs379781684	1	75,187,853	C	0.15	−19.4	3.4	7.03
Milk kg (LA3)	rs209578598	1	83,272,783	A	0.36	−349	77	7.82
Protein % (LA2)	rs876040025	3	11,592,760	C	0.05	−0.098	0.015	7.34
Protein % (LA3)	rs41586418	3	13,915,936	G	0.18	−0.068	0.012	7.18
Fat % (LAm)	rs134606936	5	90,406,099	T	0.44	−0.113	0.024	7.78
Fat % (LA1)	rs211210569	5	93,516,066	T	0.23	−0.151	0.033	11.93
Fat % (LA3)	rs209372883	5	93,518,685	C	0.21	−0.186	0.051	8.26
Fat % (LA2)	rs207994397	5	93,525,076	T	0.23	−0.226	0.043	10.91
Protein % (LA3)	rs41604619	5	95,098,733	T	0.29	0.061	0.011	8.26
Protein % (LA3)	rs382685419	6	85,371,484	T	0.50	−0.054	0.013	8.03
Protein % (LA2)	rs378558630	6	85,373,205	A	0.49	−0.055	0.011	8.47
Protein % (LA1)	rs378558630	6	85,373,205	A	0.49	−0.035	0.01	7.41
Protein % (LAm)	rs378558630	6	85,373,205	A	0.49	−0.047	0.013	7.13
Milk kg (LA3)	rs385677618	8	56534074	T	0.33	−346	114	8.04
Protein kg (LA3)	rs432948152	8	56568636	G	0.33	−8.5	3.9	6.84
Protein kg (LAm)	rs210911072	8	59917537	G	0.23	−6.4	4.6	7.53
Milk kg (LAm)	rs797297575	8	60079367	G	0.14	−280	226	8.03
Protein % (LA2)	rs211239920	10	44,746,907	T	0.34	0.054	0.015	10.48
Protein % (LA3)	rs208655317	10	44,746,980	G	0.29	0.065	0.02	8.13
Protein % (LAm)	rs208655317	10	44,746,980	G	0.29	0.075	0.021	7.95
Protein % (LA1)	rs137281406	20	33,314,537	C	0.07	−0.089	0.012	7.19
Milk kg (LA2)	rs211525696	21	66,634,034	A	0.22	338	64	7.35
Milk kg (LAm)	rs385070122	21	68,335,947	A	0.09	486	84	7.42
Fat kg (LA1)	rs42120938	27	25,539,379	A	0.42	−9.4	2.4	8.95

**Table 2:** Candidate genes in QTL regions significantly associated with milk production traits in DSN.

Top SNP	QTL Start	QTL Stop	Length	Gene Names (No. of Genes)
1:75,187,853	75,045,795	75,397,280	0.35	***FGF12*** (1)
1:83,272,783	83,066,360	83,334,688	0.27	*HTR3C*, s, *ABCC5*, ENSBTAG00000052825, *PARL*, *MAP6D1*, *YEATS2* (7)
3:11,592,760	11,579,114	11,630,952	0.05	*CD1E* (1)
3:13,915,936	13,915,389	13,919,145	0.01	*ARHGEF11* (1)
5:93,516,066	93,300,498	93,662,363	0.36	*LMO3*, ***MGST1***, *SLC15A5* (3)
6:85,373,205	84,782,659	85,442,084	0.66	ENSBTAG00000053282, ENSBTAG00000053565, ENSBTAG00000015047, ENSBTAG00000004040, *SULT1B1*, ENSBTAG00000038214, *SULT1E1*, ***CSN1S1*** (8)
8:56,534,074	55,472,873	56,898,488	1.43	***TLE4*** (1)
8:60,079,367	59,912,308	60,102,002	0.19	*GBA2, RGP1, MSMP, NPR2, SPAG8, HINT2, FAM221B, TMEM8B, OR13E1, OR13E10, OR13J1C, OR13J1F* (12)
10:44,746,907	43,852,505	47,828,666	3.98	*TRIM9*, ENSBTAG00000053552, *TMX1*, *FRMD6*, *GNG2*, *RTRAF*, *NID2*, ENSBTAG00000001423, *PTGDR*, *PLEKHO2*, *PIF1*, *RBPMS2*, *OAZ2*, *ZNF609*, *TRIP4*, ENSBTAG00000049725, *PCLAF*, *CSNK1G1*, ENSBTAG00000049412, *PPIB*, *SNX22*, *SNX1*, *CIAO2A*, *DAPK2*, *HERC1*, ENSBTAG00000052707, ENSBTAG00000051076, ENSBTAG00000054388, ENSBTAG00000050908, ENSBTAG00000019474, *FBXL22*, *USP3*, *CA12*, *APH1B*, *RAB8B*, *RPS27L*, *LACTB*, *TPM1*, ENSBTAG00000040590, *TLN2* (40)
20:33,314,537	33,313,093	33,329,684	0.02	*C6* (1)
21:66,634,034	66,620,652	66,634,034	0.01	*-* (0)
21:68,335,947	68,328,315	69,356,368	1.03	*ZFYVE21*, *PPP1R13B*, *ATP5MJ*, *TDRD9*, *RD3L*, *ASPG*, *KIF26A*, *C21H14orf180*, *TMEM179*, ENSBTAG00000054250, ENSBTAG00000007187, *ADSS1*, *SIVA1*, *AKT1*, *ZBTB42*, *CEP170B*, *PLD4* (17)
27:25,539,379	25,489,516	25,563,491	0.07	***TNKS*** (1)

For each QTL, the top SNP, QTL start and stop position, QTL length in Mb and the genes and number of genes located in the QTL region are provided. Prioritized candidate genes are marked in bold.

## Data Availability

The datasets analyzed in this study can be found in the European Nucleotide Archive (ENA) (https://www.ebi.ac.uk/ena/browser/home) (WGS data: PRJEB45822, DSN200k SNP chip data: PRJEB46861, IlluminaBovineSNP50 BeadChip data: PRJEB42513).

## Supplementary Materials

The following supporting information can be downloaded at: https://www.mdpi.com/article/10.3390/genes14030581/s1, Figure S1: Overview of inflation factors for all investigated traits plotted separately for autosomes and chromosome X; Table S1: Fixed effects contributing to GWAS models estimated using Akaike information criterion; Table S2: Full list of associated variants (*p* < 0.1) from GWAS with milk production traits in DSN; Table S3: Pairwise Pearson correlation between investigated milk performance traits; Table S4: Heritability for investigated milk production traits in DSN; Table S5: List of genes and corresponding variants in the identified QTL regions; Table S6: Results from enrichment analysis in identified QTL regions using g:Profiler; Table S7: List of publications with GWAS results overlapping with loci identified in this study.
